# Performance of the Tariff Method: validation of a simple additive algorithm for analysis of verbal autopsies

**DOI:** 10.1186/1478-7954-9-31

**Published:** 2011-08-04

**Authors:** Spencer L James, Abraham D Flaxman, Christopher JL Murray

**Affiliations:** 1 Institute for Health Metrics and Evaluation, University of Washington, 2301 Fifth Ave., Suite 600, Seattle, WA 98121, USA

**Keywords:** Verbal autopsy, validation, gold standard, Tariff Method, cause of death, mortality, cause-specific mortality fractions

## Abstract

**Background:**

Verbal autopsies provide valuable information for studying mortality patterns in populations that lack reliable vital registration data. Methods for transforming verbal autopsy results into meaningful information for health workers and policymakers, however, are often costly or complicated to use. We present a simple additive algorithm, the Tariff Method (termed Tariff), which can be used for assigning individual cause of death and for determining cause-specific mortality fractions (CSMFs) from verbal autopsy data.

**Methods:**

Tariff calculates a score, or "tariff," for each cause, for each sign/symptom, across a pool of validated verbal autopsy data. The tariffs are summed for a given response pattern in a verbal autopsy, and this sum (score) provides the basis for predicting the cause of death in a dataset. We implemented this algorithm and evaluated the method's predictive ability, both in terms of chance-corrected concordance at the individual cause assignment level and in terms of CSMF accuracy at the population level. The analysis was conducted separately for adult, child, and neonatal verbal autopsies across 500 pairs of train-test validation verbal autopsy data.

**Results:**

Tariff is capable of outperforming physician-certified verbal autopsy in most cases. In terms of chance-corrected concordance, the method achieves 44.5% in adults, 39% in children, and 23.9% in neonates. CSMF accuracy was 0.745 in adults, 0.709 in children, and 0.679 in neonates.

**Conclusions:**

Verbal autopsies can be an efficient means of obtaining cause of death data, and Tariff provides an intuitive, reliable method for generating individual cause assignment and CSMFs. The method is transparent and flexible and can be readily implemented by users without training in statistics or computer science.

## Background

Verbal autopsies (VAs) are increasingly being used to provide information on causes of death in demographic surveillance sites (DSSs), national surveys, censuses, and sample registration schemes [1-3]. Physician-certified verbal autopsy (PCVA) is the primary method used to assign cause once VA data are collected. Several alternative expert-based algorithms [4-6], statistical methods [7-9], and computational algorithms [7] have been developed. These methods hold promise, but their comparative performance needs to be evaluated. Large-scale validation studies, such as the Population Health Metrics Research Consortium (PHMRC) [10], provide objective information on the performance of these different approaches.

The main limitation to date of PCVA is the cost and feasibility of implementation. Finding and training physicians to read VAs in resource-poor settings has proven challenging, leading in some cases to long delays in the analysis of data [1,11]. In some rural areas with marked shortages of physicians, assigning the few available physicians to read VAs may have a very high opportunity cost in terms of health care delivery. Lozano et al. [12] have also shown that there is a substantial idiosyncratic element to PCVA related to physician diagnostic performance. In contrast, some automated methods (whether statistical or computational in nature) have demonstrated performance similar to PCVA [7,8], but some users may be uncomfortable with the "black box" nature of these techniques. It is often very difficult for users to unpack how decisions on a cause are reached. Furthermore, the actual statistics and mechanics that form the basis for cause assignments are difficult to access and understand due to the myriad computations involved. One method, the King-Lu method, is a direct cause-specific mortality fraction (CSMF) estimation approach [13,14] that does not assign cause to specific deaths, making it even harder for a user to understand how the cause of death is being determined.

Empirical methods that use the observed response pattern from VAs in a training dataset have an advantage over expert judgment-based methods in that they capture the reality that some household respondents in a VA interview may respond "yes" to some items even when they would not be considered part of the classical clinical presentation for that cause. For example, 43% of households report coughing as a symptom for patients who died from a fall, and 58% of households report a fever for patients who died from a road traffic accident. However, a limitation of many existing methods such as Simplified Symptom Pattern and Random Forest is that they may not give sufficient emphasis to pathognomonic signs and symptoms. For example, if 20% of patients dying of epilepsy report convulsions, and only 2% of nonepilepsy patients report convulsions, a statistical model will not assign this symptom as much significance as these data imply. Put another way, Bayesian methods such as InterVA and Symptom Pattern and statistical methods such as King-Lu direct CSMF estimation assume that the probability of signs and symptoms conditional on true cause is constant, but in reality it is not. There are subsets of patients who may have signs and symptoms that are extremely informative, and other subsets with less clearly defined signs/symptoms.

In this paper, we propose a simple additive approach using transparent, intuitive computations based on responses to a VA instrument. Our premise is that there ought to be highly informative signs or symptoms for each cause. Our goal is to develop an approach to cause of death estimation based on reported signs and symptoms that is simple enough to be implemented in a spreadsheet so that users can follow each step of cause assignment. We illustrate the development of this approach and then use the PHMRC gold standard VA validation study dataset [10] to assess the performance of this approach compared to PCVA, which is current practice.

## Methods

### Logic of the method

The premise behind the Tariff Method is to identify signs or symptoms collected in a VA instrument that are highly indicative of a particular cause of death. The general approach is as follows. A tariff is developed for each sign and symptom for each cause of death to reflect how informative that sign and symptom is for that cause. For a given death, based on the response pattern in the VA instrument, the tariffs are then summed yielding an item-specific tariff score for each death for each cause. The cause that claims the highest tariff score for a particular death is assigned as the predicted cause of death for that individual. The tariffs, tariff scores, and ranks are easily observable at each step, and users can readily inspect the basis for any cause decision.

Based on a training dataset in which the true cause is known and a full verbal autopsy has been collected, we can compute a tariff as a function of the fraction of deaths for each variable or item that has a positive response. The tariff can be thought of as a robust estimate of how different an item response pattern is for a cause compared to other causes, formally:

where tariff_ij _is the tariff for cause i, item j, x_ij _is the fraction of VAs for which there is a positive response to deaths from cause i for item j, median(x_ij_) is the median fraction with a positive response for item j across all causes, and interquartile range x_ij _is the interquartile range of positive response rates averaged across causes. Note that as defined, tariffs can be positive or negative in value. As a final step, tariffs are rounded to the nearest 0.5 to avoid overfitting and to improve predictive validity.

For each death, we compute summed tariff scores for each cause:

where x_jk _is the response for death k on item j, taking on a value of 1 when the response is positive and 0 when the response is negative, and w is the number of items used for the cause prediction. It is key to note that for each death, a different tariff score is computed for each of the possible causes. In the adult module of the PHMRC study, for example, there are 46 potential causes and so there are 46 different tariff scores based on the tariffs and the response pattern for that death. For actual implementation, we use only the top 40 items for each cause in terms of tariff to compute a tariff score. The set of 40 items used for each cause prediction are not mutually exclusive, though cumulatively across all cause predictions the majority of items in the PHMRC VA questionnaire are used for at least one cause prediction.

**Figure 1:**
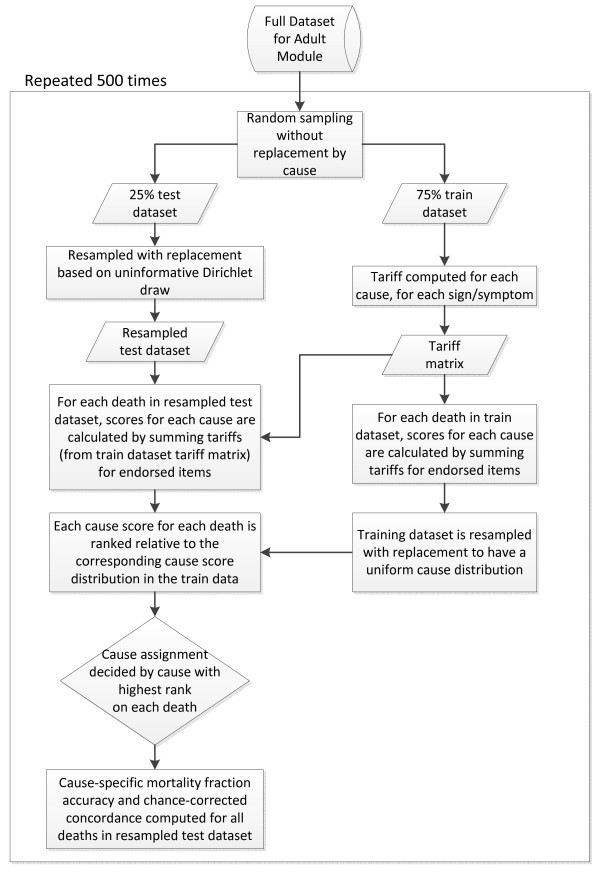
**Schematic diagram showing the process of making cause assignments starting with the full dataset**. All steps within the boxed area are repeated 500 times.

Once a set of tariff scores has been obtained for a given death, the cause of death can be assigned in several ways. The easiest method is to simply assign the cause with the highest tariff score. However, some causes may have inherently higher tariffs. To address this issue, each test death's cause-specific score is ranked in comparison to all of that cause's scores for deaths in the training dataset, which has been resampled to have a uniform cause distribution. This ranking transformation normalizes the tariff scores and draws on the information found in the training dataset. The cause that claims the highest rank on each death being tested receives the cause assignment for that death. In repeated tests, we have found the ranking transformation improves performance and is the preferred final step for assigning cause. By making cause assignments based on rank for each individual death through the use of the training dataset, we also emulate how the method could be used for individual cause assignment in the field, since cause assignment in the field would be based on ranking a single death relative to the entire validation dataset's tariff scores. This entire process is illustrated in Figure 1.

### Implementation of the Tariff Method

We use the PHMRC gold standard VA training datasets to develop tariffs and then to assess the performance of Tariff compared to PCVA. Details on the design of this multicountry study are provided elsewhere [10]. The study collected 7,836 adult, 2,075 child, and 2,631 neonatal deaths with rigorously defined clinical diagnostic and pathological criteria. For each death, the PHMRC VA instrument was applied. The resulting VA dataset consists of responses to symptoms and signs that may be expressed as dichotomous, continuous, and categorical variables. The survey instrument also included items for the interviewer to transcribe medical record text from the household and to take notes during the "open response" portion of the interview, when the respondent explains anything else that he/she feels is relevant. The text from these responses has been converted to dichotomous items. The continuous and categorical variables, such as "how long did the fever last?" were also converted to dichotomous variables. These data processing steps are described in more detail elsewhere [10]. We use the dichotomized training datasets to develop tariffs. We then compute tariff scores for each death in the test and train datasets and assign a cause of death to each death in the test dataset. We compute chance-corrected concordance and CSMF accuracy [15] on the cause of death predictions in the test dataset to avoid in-sample analysis. Chance-corrected concordance is a sensitivity assessment that measures the method's ability to correctly determine individual cause of death. CSMF accuracy is an index that measures a VA method's ability to estimate a population's cause-specific mortality fractions and is determined by calculating the sum of the absolute value of CSMF errors compared to the maximum possible error in CSMFs. Examination of the tariff score ranks can yield a second, third, etc., most likely cause of death. We also compute partial chance-corrected concordance for up to six causes [15]. We undertake separate analyses for adult, child, and neonatal deaths. It is important to note that for each train-test data split from the PHMRC study, we compute a new set of tariffs based only on that particular training set. In other words, in no case are test data used in the development of the tariff that is applied to that particular test dataset.

We have repeated the development of tariffs and tariff scores using household recall of health care experience (HCE) and excluding these variables [10] in order to estimate the method's performance in settings where access to health care is uncommon. HCE items capture any information that the respondent may know about the decedent's experiences with health care. For example, the items "Did [name] have AIDS?" or "Did [name] have cancer?" would be considered HCE items. Text collected from the medical record is also classified as HCE information. For example, the word "malaria" might be written on the decedent's health records and would be considered an HCE item. Based on the validation dataset collected by the PHMRC [10], we were able to estimate causes of death and evaluate the method for 34 causes for adults, 21 causes for children, and 11 causes for neonates. We compared Tariff's performance to PCVA for the same cause lists and item sets for the adult and child results; however, PCVA produces estimates for only six neonate causes and consequently direct comparison for neonates was not possible.

In order to analyze the performance of Tariff in comparison with PCVA across a variety of cause of death distributions, 500 different cause compositions based on uninformative Dirichlet sampling [10] were processed with both Tariff and PCVA. The frequency with which Tariff outperforms PCVA in both chance-corrected concordance and CSMF accuracy is then computed across these 500 population cause-specific constructs.

## Results

### Tariffs

Table 1 shows selected tariffs that exemplify pathological plausibility and how certain signs/symptoms are strongly predictive of certain causes as compared to other causes. For example, in predicting diabetes with skin infection, the sign of an "ulcer oozing pus" has a positive response rate frequency that is 25 interquartile ranges above the median frequency for this sign across causes. This will result in any death reporting this sign to be highly-ranked within the cause prediction scores. The word "cancer" being written on one's health care records has a relatively high tariff for both esophageal cancer and cervical cancer, demonstrating that it has predictive value despite being less specific than other items. It is interesting to note that approximately 50% of maternal hypertensive disorder deaths reported convulsions, and 50% of diabetes with skin infection deaths reported ulcer oozing pus, yet these two sign-cause combinations have markedly different tariffs. This reflects how the tariff computation can capture both the strength and uniqueness of a sign/symptom in predicting a cause. These two examples have equal strength in terms of the sign/symptom-cause endorsement rate, but the sign "ulcer oozing pus" is more unique to diabetes with skin infection than convulsions are to hypertensive disorders.

**Table 1:** Selected tariffs in the adult module of the PHMRC dataset

	Signs/Symptoms				
	
Causes	Ulcer oozed pus	Lump in the neck	Convulsions	Pain in left arm	Free text: "cancer"
**Diabetes with skin infection**	25	0.5	0.5	1	-0.5

**Esophageal cancer**	-0.5	8.5	-1.5	1	4.5

**Hypertensive disorder (maternal)**	-0.5	0.5	7	-0.5	-0.5

**Acute myocardial infarction**	0	-1	-0.5	4.5	0.5

**Cervical cancer**	0	0.5	-0.5	-0.5	7

Tariffs were calculated as explained in the Methods section. These particular tariffs were selected because they demonstrate how the method can be somewhat intuitive from a medical perspective. For example, an ulcer oozing pus is a plausible sign for a person who died of diabetes with skin infection, though it seems somewhat less plausible for someone dying of acute myocardial infarction.

Additional files 1, 2, and 3 show the tariffs (derived from the full dataset) for the top 40 items based on tariff absolute value for each cause for the adult, child, and neonate modules, respectively.

### Validation of Tariff cause assignment

#### Individual death assignment

**Figure 2:**
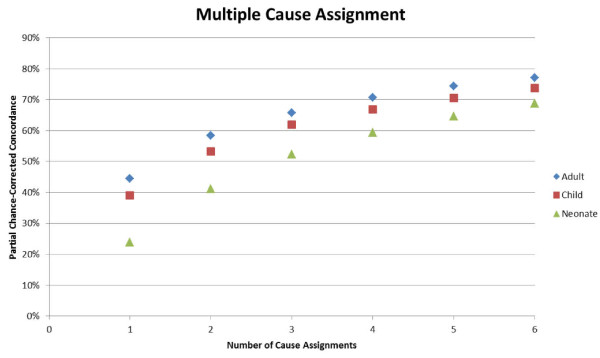
**Partial chance-corrected concordance for the adult, child, and neonate predictions for making multiple cause of death assignments for each death**. Multiple assignments can be made by looking at the top-ranked causes based on the tariff scores for each cause. For a given death, for example, AIDS, TB, and pneumonia might be the three most likely causes of death, thus improving the probability that one of those causes is correct. The partial chance-corrected concordance calculation includes a correction term to compensate for the inherently higher probability of making a correct assignment when multiple causes are assigned.

Table 2 compares overall median chance-corrected concordance across 500 train-test data splits for Tariff and PCVA for adults, children, and neonates. Among adults, Tariff outperforms PCVA when health care experience is excluded and is not significantly different than PCVA when health care experience information is included. PCVA outperforms Tariff in chance-corrected concordance for the child module both with and without health care experience information. Tariff achieves 21.6% (without HCE) and 23.9% (with HCE) chance-corrected concordance in the neonate module analysis. Neonate results between Tariff and PCVA are not directly comparable because PCVA cannot predict causes of death for all 11 neonate causes and consequently aggregates the five premature delivery causes into a single premature delivery cause. Figure 2 provides details on how well Tariff identifies the true cause as the second, third, fourth through to sixth cause in the list. For all age groups, partial chance-corrected concordance increases steadily as extra causes are considered on the list. It is important to note that partial chance-corrected concordance includes a correction factor for concordance due to chance. Tariff achieves 66% partial chance-corrected concordance if three cause assignments are made for adults, 62% for children, and 52% for neonates.

**Table 2:** Median chance-corrected concordance (%) for Tariff and PCVA with 95% uncertainty interval (UI), by age group with and without HCE information

		Tariff		PCVA	
		
		Median	95% UI	Median	95% UI
**Adult**	**No HCE**	34.3	(34.1, 34.5)	29.7	(29.4, 29.8)
	
	**HCE**	44.5	(44.2, 44.7)	44.6	(44.3, 44.8)

**Child**	**No HCE**	28.8	(28.4, 29.2)	36.3	(35.9, 36.6)
	
	**HCE**	39.0	(38.4, 39.4)	47.8	(47.1, 48.3)

**Neonate**	**No HCE**	21.6	(21.2, 22.2)	27.6	(27.2, 28.0)
	
	**HCE**	23.9	(23.6, 24.4)	33.3	(32.8, 33.7)

Tariff outperforms or matches the performance of PCVA in the adult module, while PCVA outperforms Tariff in the child module. Results are not comparable for neonates since PCVA can only make cause assignments on six neonate causes, while Tariff makes cause assignments on 11 neonate causes.

**Figure 3:**
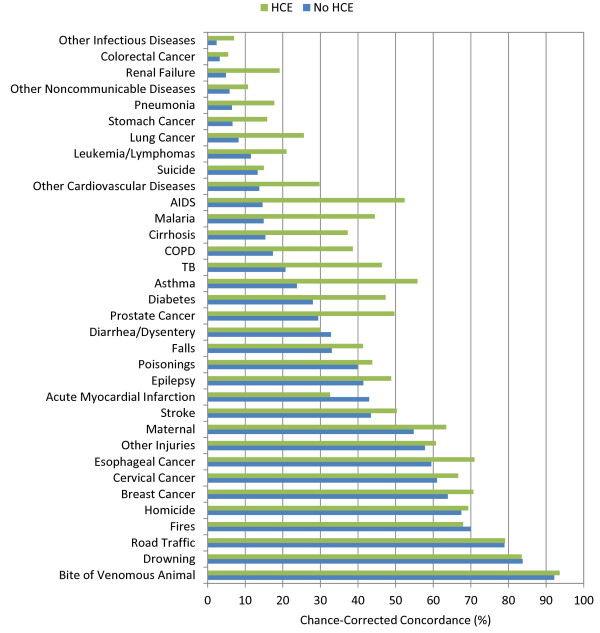
**Median chance-corrected concordance (%) across 500 test splits, by adult cause with and without HCE**.

**Figure 4:**
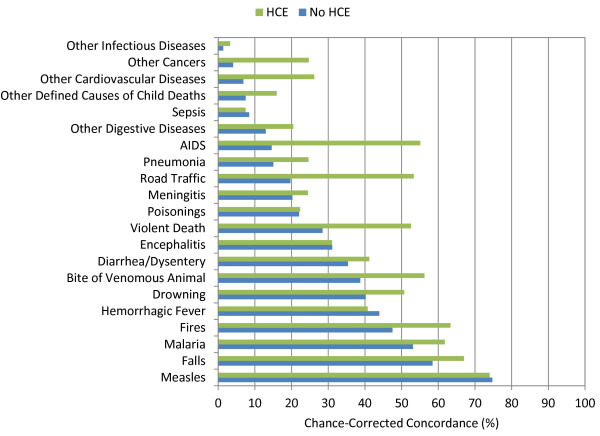
**Median chance-corrected concordance (%) across 500 test splits, by child cause with and without HCE**.

**Figure 5:**
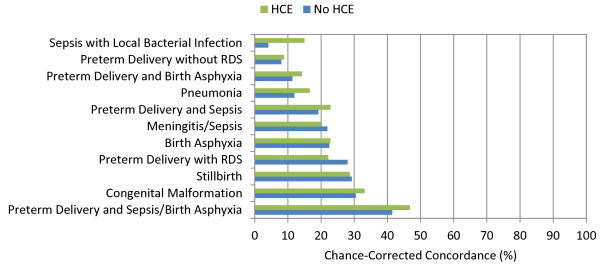
**Median chance-corrected concordance (%) across 500 test splits, by neonate cause with and without HCE**.

**Figure 6:**
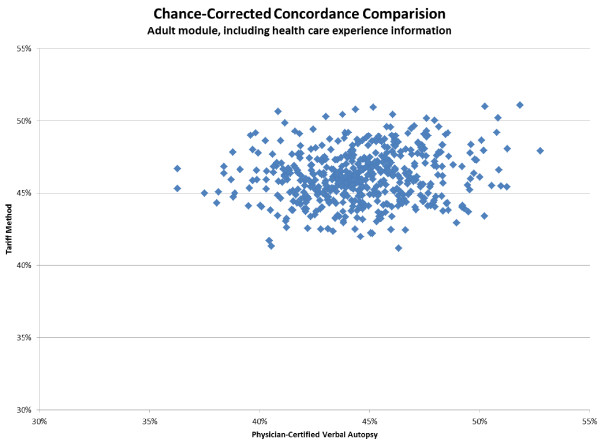
**Chance-corrected concordance comparison scatter for 500 splits of PCVA and Tariff adult module estimations**. These results included the use of HCE information.

Additional file 4 provides cause-specific chance-corrected concordances for Tariff. For adults, when excluding household recall of health care experience, Tariff yields median chance-corrected concordances over 50% for a number of injuries, including bite of venomous animal, breast cancer, cervical cancer, drowning, esophageal cancer, fires, homicide, maternal, other injuries, and road traffic. Addition of health care experience raises chance-corrected concordance over 50% for AIDS, asthma, and stroke. Additional file 4 also shows that in children without household recall of health care experience, median chance-corrected concordance is over 50% for falls, malaria, and measles. With HCE, the list expands to also include AIDS, bite of venomous animal, drowning, fires, road traffic, and violent death. In neonates, the best performance for Tariff is for preterm delivery and sepsis/birth asphyxia, preterm delivery with respiratory distress syndrome, congenital malformation, and stillbirth. Figures 3, 4, and 5 show visual comparisons of each cause-specific chance-corrected concordance with and without HCE for adults, children, and neonates, respectively. These figures also highlight the value of adding HCE information and demonstrate how individual cause assignment is difficult for certain causes when HCE information is not available. For example, the important adult causes of AIDS, malaria, and TB have low concordance when HCE information is withheld, though performance does improve dramatically when HCE information is added. Similarly, chance-corrected concordance improves roughly four-fold for AIDS in the child module when HCE is added. Figure 6 shows a comparison for adults with HCE of concordance achieved with Tariff and PCVA applied to the same 500 test datasets. These results show that PCVA varies more than Tariff in chance-corrected concordance, despite their median across 500 splits being approximately the same.

#### CSMF estimation

To estimate Tariff's ability to accurately determine CSMFs, we predicted causes of death for 500 different test datasets with varying cause compositions. Table 3 shows that Tariff yields more accurate estimates of CSMFs than PCVA for adults and children, both with and without health care experience information. Since PCVA cannot make cause assignments on the full list of 11 neonate causes, it is not possible to directly compare PCVA and Tariff in accuracy.

**Table 3:** Median CSMF accuracy for Tariff and PCVA with 95% UI, by age group with and without HCE information

		Tariff		PCVA	
		
		Median	95% UI	Median	95% UI
**Adult**	**No HCE**	0.695	(0.690, 0.699)	0.624	(0.619, 0.631)
	
	**HCE**	0.745	(0.739, 0.753)	0.675	(0.669, 0.680)

**Child**	**No HCE**	0.642	(0.635, 0.651)	0.632	(0.626, 0.642)
	
	**HCE**	0.709	(0.704, 0.715)	0.682	(0.671, 0.690)

**Neonate**	**No HCE**	0.663	(0.655, 0.671)	0.695	(0.682, 0.705)
	
	**HCE**	0.679	(0.670, 0.689)	0.733	(0.719, 0.743)

PCVA results for neonates are shown at a six-cause level, since analysis was not possible at the same 11-cause level as Tariff.

**Figure 7:**
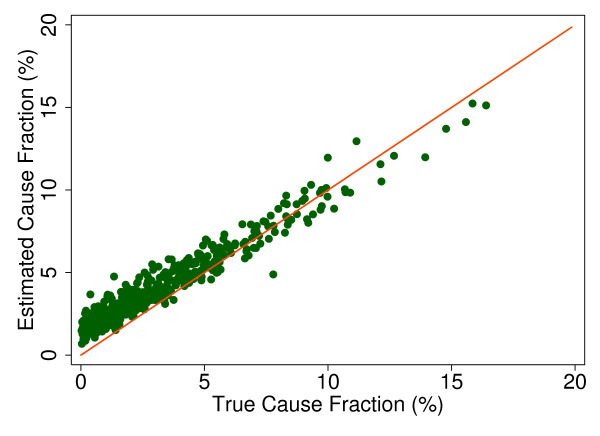
**True versus estimated mortality fractions for drowning, adult module with HCE information**.

**Figure 8:**
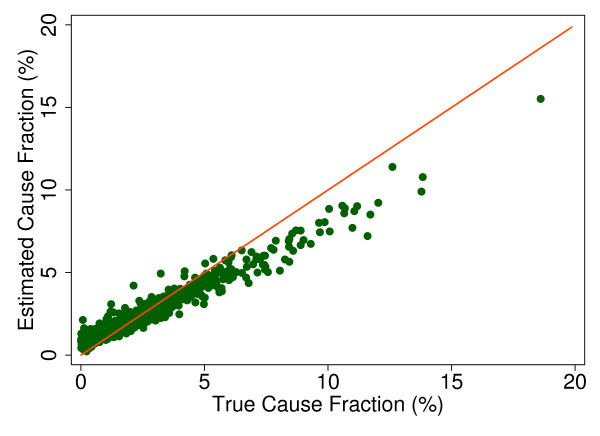
**True versus estimated mortality fractions for breast cancer, adult module with HCE information**.

**Figure 9:**
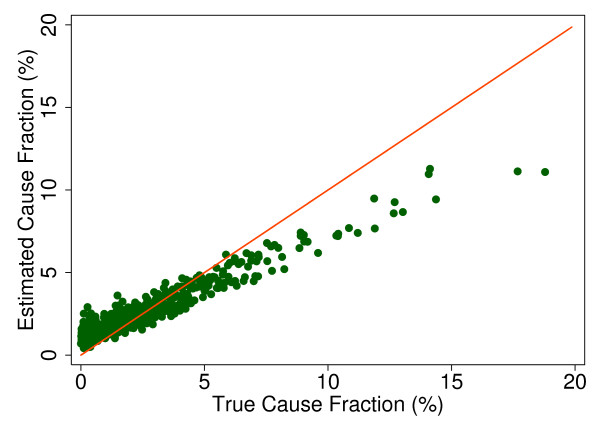
**True versus estimated mortality fractions for maternal causes, adult module with HCE information**.

**Figure 10:**
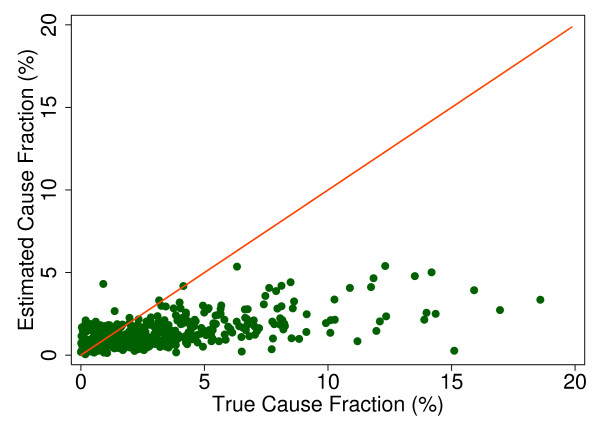
**True versus estimated mortality fractions for stomach cancer, adult module with HCE information**.

Additional file 5 shows the slope, intercept, and root mean squared error (RMSE) of regressing the estimated CSMF as a function of true CSMF for all causes across 500 test splits. We have selected four adult causes based on Additional file 5 to illustrate a range of cases where Tariff produces good to relatively poor estimates of the CSMF as a function of the true CSMF. Figure 7 shows the estimated CSMF for drowning compared to the true CSMF for drowning in adults across 500 test datasets. In general, across a wide range of true CSMFs, Tariff performs well in estimating the CSMF from this cause. This quality is further evidenced by the results from the regression. Drowning has an intercept of 1.5%, which means that even if there are no true deaths from drowning in a VA dataset, Tariff will tend to predict a CSMF of approximately 1.5%. However, the slope of 0.817 and the RMSE of 0.006 also indicate that estimations tend to track the true CSMFs fairly closely, and that estimated CSMFs will not vary widely for a given true CSMF. For breast cancer, shown in Figure 8, Tariff can accurately determine the mortality fractions in test splits with small to modest numbers of true breast cancer deaths; however, in test splits with high breast cancer mortality fractions, Tariff tends to underestimate the fraction. The results from the regression for breast cancer show that estimates are slightly less noisy than for drowning and that the method will start to systematically underestimate CSMFs beyond a true CSMF of approximately 2.5%. Figure 9 shows the same relationship for maternal, with a slightly higher threshold for when the method begins to underestimate CSMFs. In this case, however, while there is still a generally good relationship between the true and estimated CSMFs, at low true CSMFs Tariff tends to overestimate the cause fraction, while at very high CSMFs, it has a slight tendency to underestimate. At the other end of the spectrum, Tariff does a poor job of estimating the population fraction of deaths due to stomach cancer, shown in Figure 10, and tends to underestimate the true cause fraction above 2%. The RMSEs provide a measure of the noise or precision in each cause's predictions. In the adult predictions including the use of HCE information, the RMSE ranged from 0.005 for maternal causes to 0.019 for other noncommunicable diseases.

**Figure 11:**
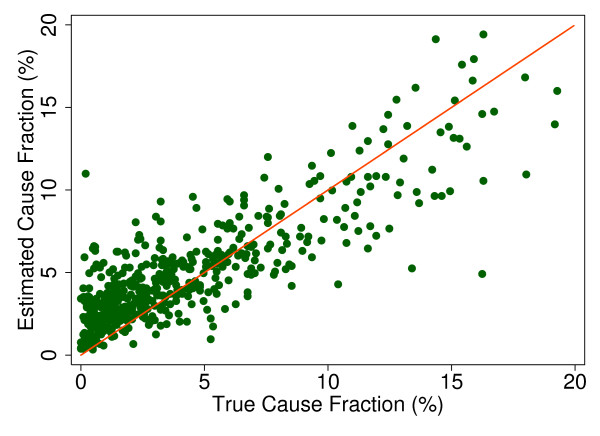
**True versus estimated mortality fractions for measles, child module with HCE information**.

**Figure 12:**
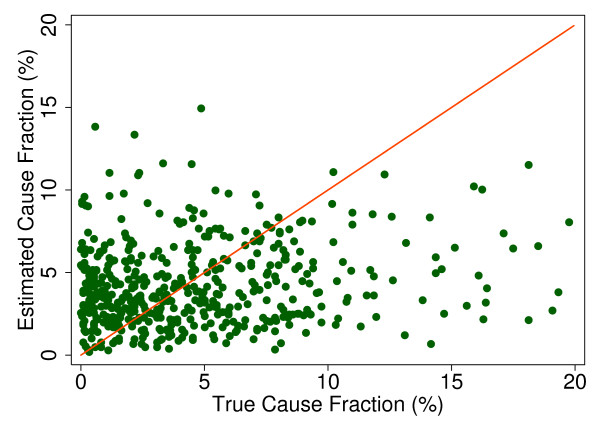
**True versus estimated mortality fractions for sepsis, child module with HCE information**.

We performed similar analyses for the child and neonate results (full regression results also shown in Additional file 5). Figure 11 demonstrates how Tariff tends to overpredict measles CSMFs in populations with a smaller measles fraction. As the true measles fraction increases, however, Tariff does not systematically over- or underestimate the mortality fractions to the extent seen in other causes. Furthermore, the estimates for measles CSMF in children are much noisier than other examples for adults. This quality is also evidenced by the higher RMSE of 0.019. For child sepsis, in contrast, Tariff tends to underestimate CSMFs as the true cause fraction increases. The true versus estimated sepsis CSMFs are shown in Figure 12. The RMSEs for children are higher than for adults, ranging from 0.013 for road traffic accidents to 0.033 for malaria.

**Figure 13:**
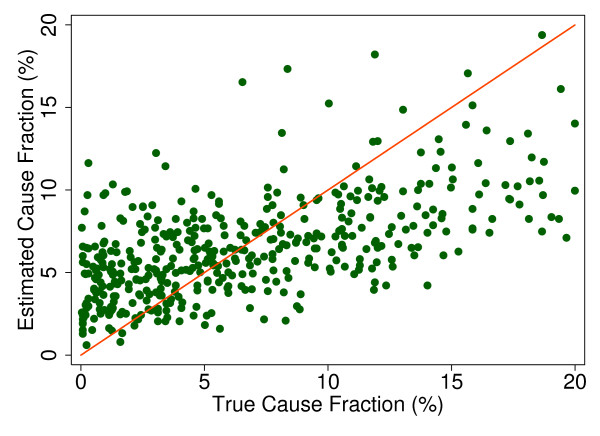
**True versus estimated mortality fractions for congenital malformation, neonate module with HCE information**.

**Figure 14:**
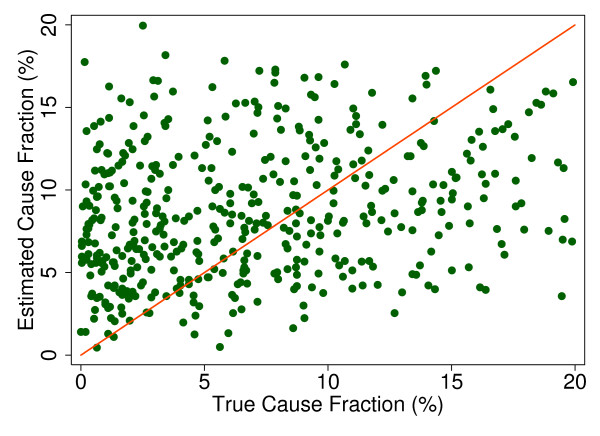
**True versus estimated mortality fractions for preterm delivery with respiratory distress syndrome, neonate module with HCE information**.

The neonate CSMF estimation tends to differ from the true cause fraction more frequently than for child or adult deaths. Congenital malformation, shown in Figure 13, exemplifies a cause for which Tariff can roughly determine the correct CSMF regardless of the true CSMF size. However, other neonatal causes such as preterm delivery with respiratory distress syndrome are subject to much noisier estimates, as shown in Figure 14. These results are further reflected in the corresponding coefficients and intercepts seen in Additional file 5, which allow for assessment of the relationship between true and estimated CSMFs. As for adults and children, the RMSE from these regressions indicate which causes can be estimated with greater precision, even if the estimation is systematically high or low. In the neonate results including the use of HCE information, the RMSE ranged from a low of 0.023 for stillbirths to 0.051 for preterm delivery and birth asphyxia and for preterm delivery, sepsis, and birth asphyxia.

## Discussion

The Tariff Method is a simple additive approach based on identifying items in a VA interview that are indicative of particular diseases. It is based on the premise that individual items or signs/symptoms should be more prominently associated with certain causes (the "signal") compared with others (the "noise"). This simple approach performs as well as or better than PCVA for adult causes in assigning an underlying cause of death, though PCVA performs better in this comparison for child deaths. At the level of particular causes, Tariff has higher chance-corrected concordances than PCVA for 14/34 adult and 8/21 child causes. Results for neonatal deaths are not comparable due to differences in cause lists. For estimating CSMFs, Tariff performs better than PCVA for adult and child deaths in all comparisons with and without household recall of health care experience. In all comparable cases, Tariff yields higher median CSMF accuracy than PCVA. Overall, at the individual and the CSMF level, Tariff in general offers a competitive alternative to PCVA. Performance for assigning neonatal causes of death, however, is worse than for PCVA.

The tariffs for each cause-item pair have already been established using Stata code, which will be available online. Using this pre-existing tariff matrix, the Tariff Method requires only multiplication and addition to make cause of death assignments for each individual death in a given dataset. Though we processed VA response data to develop our method, users need not conduct additional processing to use Tariff since our processing steps can be integrated into the code that makes cause of death assignments. The absence of a statistical model or complex computational algorithm means that the steps involved in assigning cause of death to a particular death can be completed in a spreadsheet and are readily available for user scrutiny. Further, the tariff matrix and algorithm can be implemented on a simple device such as a cell phone - the Open Data Kit research team at the University of Washington has already implemented the tariff algorithm on an Android cell phone using their Free/Libre Open-Source Survey Platform. In other words, tariff-based cause assignments can be made immediately after data collection in the field.

One of the key strengths of Tariff is its flexibility. Each item's tariff for a cause is computed independently from all other items. Consequently, any instrument's verbal autopsy items that can be mapped to one of the items in the PHMRC dataset can be evaluated using Tariff. Other methods, such as Random Forest and Simplified Symptom Pattern, require the testing data to have the same item set as the data on which the model was trained. This is an important asset of Tariff because it allows users to implement the method without having to recalculate tariffs or revise the algorithm. It can essentially be used as is for any verbal autopsy instrument with overlapping items with the PHMRC instrument.

Tariff does not take into account the interdependencies of signs and symptoms conditional on particular causes. It does not take into account the complex time sequence captured in open narratives, which are often used by physicians. How can such a simple algorithm be more effective than physicians? The answer may lie in the key attributes of Tariff that distinguish it from other methods: identification of items that are unusually important for different causes through computation of the tariff and the additive rather than multiplicative nature of the tariff score. The tariffs focus attention on the specific subset of items that are most strongly related to a given cause. The additive approach may make Tariff more robust to measurement error either in the train or test datasets.

Because of its simplicity, we plan to make available several different platforms on which to apply Tariff. Programs in R, Stata, and Python will be available for assigning a cause for a given death or set of deaths, as well as a version of Tariff in Excel for users without training in statistics packages. Tariff will also be available in the Open Data Kit for use on the Android operating system for cell phones and tablets. We hope these tools will lead to widespread testing and application of Tariff. The full sign/symptom-cause tariff matrix will also be available for user inspection and application to other verbal autopsy diagnostic methods such as Random Forest and Simplified Symptom Pattern, which rely on tariffs to identify meaningful signs and symptoms. The tariffs can also be used to refine further verbal autopsy instruments, possibly in reducing the number of survey items, since they show which specific signs/symptoms should be included for accurately predicting certain causes of death. For example, one strategy for item reduction would be to drop items that have low tariffs for all causes and then assess the change in CSMF accuracy or chance-corrected concordance when cause assignment is undertaken with the restricted item set.

Given that PCVA can be costly and time consuming, it would seem that Tariff provides an attractive alternative. Compared to the current version of InterVA [16], Tariff performs markedly better. We believe that users interested in rapid, low-cost, easy-to-understand VA methods should consider Tariff. As indicated by analysis of CSMF accuracy and true versus estimated CSMF regressions, there are certain cases where Tariff may overestimate or underestimate CSMFs for particular causes. It will be important for users of Tariff to understand these limitations, particularly for the purposes of using Tariff to better inform public health decision-making. Future research may yield new techniques to more accurately determine CSMFs based on verbal autopsy through back calculation. Tariff is also attractive to those who wish to examine the exact computation by which a verbal autopsy algorithm makes a cause of death assignment. In the future, as more gold standard deaths are collected to augment existing causes in the PHMRC dataset, or for new causes, it will be straightforward to revise existing tariffs or report tariffs for new causes. This step is particularly easy compared to other computer-automated methods, for which expansion with more causes requires revision of the algorithm itself.

## Conclusion

Verbal autopsies are likely to become an increasingly important data collection platform in areas of the world with minimal health information infrastructure. To date, methods for evaluating verbal autopsies have either been expensive or time-consuming, as is the case with PCVA, or they have been computationally complex and difficult for users to implement in different settings. This has inhibited the widespread implementation of verbal autopsy as a tool for policymakers and health researchers. Tariff overcomes both of these challenges. The method is transparent, intuitive, and flexible, and, importantly, has undergone rigorous testing to ensure its validity in various settings through the use of the PHMRC verbal autopsy dataset. Using the method on verbal autopsies to determine both individual-level cause assignment and cause-specific mortality fractions will greatly increase the availability and utility of cause of death information for populations in which comprehensive and reliable medical certification of deaths is unlikely to be achieved for many years to come, but is urgently needed for health policies, programs, and monitoring progress with development goals.

## Abbreviations

CSMF: cause-specific mortality fraction; HCE: health care experience; PCVA: physician-certified verbal autopsy; RMSE: root mean squared error; VA: verbal autopsy

## Competing interests

The authors declare that they have no competing interests.

## Authors' contributions

SLJ, ADF, and CJLM conceptualized the method and algorithm. SLJ performed analyses and helped write the manuscript. CJLM drafted the manuscript and approved the final version. CJLM accepts full responsibility for the work and the conduct of the study, had access to the data, and controlled the decision to publish. All authors have read and approved the final manuscript.

## Supplementary Material

Additional file 1 **Top 40 signs/symptoms based on absolute value tariffs for each cause in the adult module**. These tariffs were calculated using the formula provided in the Methods section. Click here for file

Additional file 2 **Top 40 signs/symptoms based on absolute value tariffs for each cause in the child module**. These tariffs were calculated using the formula provided in the Methods section. Click here for file

Additional file 3 **Top 40 signs/symptoms based on absolute value tariffs for each cause in the neonate module**. These tariffs were calculated using the formula provided in the Methods section. Click here for file

Additional file 4 **Median chance-corrected concordance (%) across 500 Dirichlet splits, by age group and cause with and without HCE**. Click here for file

Additional file 5 **Slope, intercept, and RMSE from linear regression of estimated versus true CSMFs, by age group and cause with and without HCE**. Click here for file
